# Sexual Development of *Silba adipata* (Diptera: Lonchaeidae): Effects of Diet, Ultraviolet Light and Fig Latex

**DOI:** 10.3390/insects16050495

**Published:** 2025-05-05

**Authors:** Ricardo Díaz-del-Castillo, Guadalupe Córdova-García, Diana Pérez-Staples, Andrea Birke, Trevor Williams, Rodrigo Lasa

**Affiliations:** 1 Instituto de Ecología AC (INECOL), Xalapa 91073, Veracruz, Mexico; ricardo.diaz@posgrado.ecologia.edu.mx (R.D.-d.-C.); andrea.birke@inecol.mx (A.B.); 2 Instituto de Investigaciones en Inteligencia Artificial (IIIA), Universidad Veracruzana, Xalapa 91097, Veracruz, Mexico; azolla29@gmail.com; 3 Instituto de Biotecnología y Ecología Aplicada, Universidad Veracruzana, Xalapa 91090, Veracruz, Mexico; diperez@uv.mx

**Keywords:** hydrolyzed protein, longevity, ovary, sexual maturation, testes

## Abstract

The black fig fly, *Silba adipata* McAlpine (Diptera: Lonchaeidae), is a monophagous invasive pest of fig crops. Its recent detection in Mexico has highlighted the urgent need for control strategies. However, efforts to study and manage this pest are constrained by a limited understanding of its basic biology and an inability to rear this insect in the laboratory. Some species of flies are reproductively immature at adult emergence and require specific nutrients for the development of reproductive structures. Given this, we examined the development of ovaries and testes in response to different adult diets, ovary maturation in relation to access to figs and ultraviolet (UV) light exposure and behavioral responses to fig latex. Dietary protein (hydrolyzed yeast) was essential for egg maturation. The highest prevalence of sexual maturity in females was observed at age 21 days and was not influenced by UV light or access to figs. Male testes size decreased over time irrespective of the adult diet. The consumption of latex increased when protein was not available, although the sexes differed in their response to latex over time. These findings help overcome a critical barrier to the laboratory colonization of *S. adipata* by demonstrating that protein-supplemented diets are essential for sexual maturation. However, the lack of information on the specific role of fig latex in the diet and the absence of sexual behavior during the experiments highlight key knowledge gaps. Future research should focus on identifying those stimuli that promote copulation and oviposition to understand the complete life cycle of this pest under controlled conditions.

## Simple Summary

The black fig fly, *Silba adipata,* is a lonchaeid fly that exclusively develops in figs. It has recently been detected in Mexico as an invasive exotic pest that requires effective control strategies. The basic biology of the fly is poorly understood due to an inability to reproduce this insect under controlled conditions. In the present study, we examined the effects of adult diet, access to figs and fig latex and ultraviolet light on the sexual development of female flies. Dietary protein was essential for the development of eggs, whereas exposure to ultraviolet light or figs was not influential. Testes size in males decreased over time irrespective of the adult diet. The consumption of fig latex increased when protein was not available but males and females differed in their attraction and feeding response to latex as they aged. These results should assist in the laboratory colonization of this insect by demonstrating that protein-supplemented diets are essential for sexual development. The role of latex feeding on fly reproduction and the absence of sexual behavior are clear knowledge gaps that require additional study.

## 1. Introduction

The black fig fly, *Silba adipata* McAlpine, 1956 (Diptera: Lonchaeidae), is one of the most important pests of fig (*Ficus carica* L. 1753) production. Originally native to Europe and the Middle East, this insect has become an invasive pest in South Africa [1], the United States [2] and Mexico [3]. In Mexico, the pest was first recorded in 2020 in the state of Morelos [4] and has since been reported in six other states in the center of the country [5], and most recently in the state of Veracruz on the Gulf coast of Mexico [6].

This pest is monophagous and oviposits exclusively in immature figs. Eggs are deposited in small groups beneath the scales of the ostiole, from where larvae hatch and burrow into the developing fig [7]. Information on the biology of the pest is sparse [7,8,9], especially details concerning its reproductive biology and sexual behavior. As far as we know, it has not been possible to reproduce this insect under laboratory conditions. Consequently, the reproduction and sexual development of the pest remain largely unstudied.

In anautogenous insects, egg production depends on the female’s ability to consume specific nutrients that favor ovarian development [10,11]. For anautogenous fruit flies (Diptera: Tephritidae), adult consumption of protein is critical for female sexual maturation [11,12,13] and male sexual performance [14,15]. Sexual development in most Tephritoidea flies (Lonchaeidae and Tephritidae) is gradual and varies across genera, from less than a week in *Ceratitis*, to one or two weeks in most species of *Anastrepha* and even longer in some *Bactrocera* species [16]. Longevity is closely related to sexual maturation. Species that reach sexual maturity earlier tend to have shorter lifespans, and the artificial acceleration of sexual maturation results in reduced adult longevity [17,18].

Additional factors, such as the presence of specific host plant compounds, can also favor sexual development. In the case of *S. adipata*, a strong attraction to the latex released from figs has been reported [7,19,20]. Fig latex is an aqueous suspension with antioxidative and anti-microbial properties that is released from damaged plant tissues to prevent infection by pathogens and to reduce herbivory [21]. The reason for the attraction to fig latex in *S. adipata* is unclear, although it may be linked to specific nutritional or chemical cues that promote sexual development, reproduction or survival, as has been observed in other insect–plant interactions involving host-derived compounds [22]. Previous studies have also reported an attraction to hexanol, a compound often released from ripe fruit [23]. Feeding on sugar exudates from ripe figs attached to the tree has also been reported in *S. adipata* [7,9] and feeding on a homogenate of figs, sugar and water has been reported to ensure mating [19], but mating behavior, courtship and copulation have not been observed.

An additional factor that can affect the physiology and feeding behavior of insects is their exposure to ultraviolet (UV) light [10,24]. Laboratory illumination is usually designed to emit light in the visible spectrum (400–700 nm) with little if any light emitted in the UV-A section of the spectrum (315–400 nm). As *S. adipata* is native to the Mediterranean and Middle East where the intensity of solar radiation is high, it may be that UV-A light could play a role in the sexual development of this fly.

In the present study, we provided a comprehensive description of the reproductive organs of male and female *S. adipata* and examined the influence of age, diet, access to fig latex and figs and the effect of UV-A light exposure on ovary development under laboratory conditions. We also assessed the effect of diet and age on testes development. Finally, we examined latex feeding behavior and the adult longevity of both sexes. This study highlights the importance of dietary protein in the sexual development of female flies and examines the role of fig-derived compounds on the reproductive biology of this little-studied invasive pest.

## 2. Materials and Methods

### 2.1. Collection and Handling of Samples

Infested figs were collected in late October 2023 from plantations located adjacent to the villages of Tatatila (19°41′27″ N; 97°6′38″ W, 2046 m elevation), La Ermita (19°41′21″ N; 97°6′50″ W, 2132 m elevation) and Tenexpanoya (19°39′38″ N; 97°8′35″ W, 1840 m elevation), in the state of Veracruz, Mexico. Figs, believed to be Brown Turkey cultivar, were sampled from trees or occasionally from the ground beneath trees. Only figs in a phenological stage between 71 and 75 on the BBCH scale [25], with no signs of decomposition, were selected.

In the laboratory, figs were placed in open plastic containers (30 × 20 × 15 cm) with a 5 mm layer of vermiculite placed over a paper towel for pupation. Each container was placed in a home-made cage (60 × 60 × 90 cm) of mosquito netting on a plastic frame. Adult flies that emerged were collected from cages daily and temporarily placed (less than 3–4 h) in plastic cages (3000 cm^3^ capacity) with a source of refined sugar and a tube of water. During this period, flies were identified by their morphological characteristics [26] and were then transferred to 500 cm^3^ cup-cages (10 to 15 flies per cage) prior to their use in experiments. Flies that were not identifiable as *S. adipata* were discarded.

All the figs and adult flies were maintained in a controlled temperature laboratory at 24 ± 1 °C, 65 ± 10% relative humidity with a 12 h:12 h photoperiod provided by LED strip lights (3500–4500 lux) measured using a photometer (YK-10 LX, LT Lutron, Taipei, Taiwan). All the experimental procedures described in the following sections were performed under these conditions unless otherwise stated.

### 2.2. Reproductive System of S. adipata

A total of eight males and eight females (aged 15–21 d) were selected and sexed based on the shape of the tip of the abdomen. These individuals were used to describe and measure the genitalia and reproductive organs. During this period, flies were maintained together in cages (3000 cm^3^ capacity) with a water source and continuous access to sugar and hydrolyzed brewer’s yeast (MP Biomedicals, Solon, OH, USA) as a protein source. Each adult was dissected and photographed under a binocular microscope (Leica EZ4, Leica Microsystems, Deerfield, IL, USA) at a magnification of ×35 and an image size of 2048 × 1536 pixels and 96 ppi resolution. The cross-sectional area of different organs such as ovaries, oocytes and testes, was measured from images of each insect using ImageJ software ver. 1.54 [27]. The images of the sexual organs were edited with Pixlr [28] to remove glare and appropriately represent each of the structures. Enlarged images of the signum and the ejaculatory bulb were captured using a camera mounted on an Axio Lab A1 compound microscope (Carl Zeiss Microscopy, Oberkochen, Germany) at 10× magnification and a Leica S8 APO microscope (Leica Microsystems, Wetzlar, Germany) at 80× magnification, respectively. The mean cross-sectional area of both ovaries and both testes was calculated for each insect and at least three oocytes were measured per female to obtain a mean value.

### 2.3. Effect of Sugar, Protein and Fig Latex Ingestion on Sexual Development

To determine the effects of adult diet on sexual development, male and female flies were separated immediately following emergence into single-sex groups of 10–15 flies. Each group was placed in a transparent plastic cage (3000 cm^3^ capacity, 23.5 × 13.5 × 13.5 cm) that was sealed with a nylon mesh lid for ventilation. Flies were offered one of the following four diets: (i) refined sugar, (ii) refined sugar + hydrolyzed protein, (iii) refined sugar + fig latex, (iv) refined sugar + hydrolyzed protein + fig latex. Sugar was included in all treatments to promote fly survival given the extended period over which sexual development occurs in this species. Each solid component of the diet (sugar and protein) was available continuously in separate vials placed at the base of the cage along with a vial of water with a cotton wick. Diet components were not mixed to provide free access to both. Latex was provided by cutting the stems and leaves of fig plants and, by using a micropipette, a 5 µL droplet of latex was transferred to the wall of the cup cage for fly consumption every day. As a source of latex, fig plants of ~50 cm height were grown in pots of soil in an experimental greenhouse.

Flies of both sexes (8–15 females, 8–10 males) were randomly selected from eight different cages per diet (replicates) at 3, 6, 9, 15, 21 and 27 days after the start of the experiment. The flies were sacrificed at −20 °C and placed in 70% ethanol until examination. As a control, groups of 10 flies of both sexes that had recently emerged (<24 h post-emergence) were sacrificed and stored in 70% ethanol. Cages were only sampled once and never re-sampled. Prior to dissection, flies were removed from 70% ethanol separated according to sex and rehydrated in deionized water for 1 h. Each fly was then dissected in physiological saline and the ovaries and testes were photographed as described in Section 2.2. The insect’s head was also photographed and measured at its widest point across the eyes as an indicator of adult body size [29].

### 2.4. Effects of Exposure to Ripe Fruit on Female Sexual Development

Following the procedures described in Section 2.3, groups of 10–15 newly emerged female flies were placed in cages (3000 cm^3^ capacity) and given access to a water source and one of the following treatments: (i) refined sugar + hydrolyzed protein, (ii) refined sugar + hydrolyzed protein + ripe figs. Figs were cut longitudinally and opened to expose the flesh. Figs were replaced at 3-day intervals. Groups of 8–10 randomly selected flies were collected from 10 different cages at 6, 9, 15 and 21 days after the start of the experiment, stored in 70% ethanol and subsequently rehydrated for 1 h, dissected and photographed for ovary measurement as described in Section 2.3. Cages of the two treatments were placed at distance of 20–30 cm from one another.

### 2.5. Effect of Ultraviolet Exposure on Female Sexual Development

As adult female flies are maintained in the laboratory under LED visible light, the possible influence of UV-A exposure on sexual development was tested as follows. Groups of 10–15 recently emerged female flies (<24 h old) were placed in cup-cages with continuous access to a water source and sugar + hydrolyzed protein as food. Cages were placed in a ventilated cardboard box and illuminated from above using either (i) 9W LED strip light (model Q09-73D, QOP Iluminación, Mexico City, Mexico) placed 20 cm above cup-cages or (ii) the same 9W LED strip light supplemented with two UV-A LED bulbs, 400 nm peak emission (Lummi model 713068, Importaciones Mustri, Naucalpan de Juárez, Mexico), placed at either end of the strip light, also 20 cm above the cup-cages. The intensity of light at the roof of the cup-cages was similar in both treatments (2950–3150 lux) and temperature, measured using an Onset Hobo datalogger (Hobo Data Loggers, Bourne, MA, USA), did not vary significantly in either setting (23.96–23.98 °C). The photoperiod was 12 h:12 h in both treatments. Eight to 10 female flies were collected from four different cages per treatment after 9, 15 and 21 d once the experiment started, placed in 70% ethanol and later dissected and photographed for ovary measurement as described in Section 2.3.

### 2.6. Male and Female Responses to Fig Latex

This experiment aimed to compare age and dietary effects on male and female latex attraction and consumption. For this, groups of 40 recently emerged flies (20 males + 20 females) were placed in cages of 3000 cm^3^ capacity with a water source. Access to the interior was provided through a front gauze mesh for safe handling. Flies were continuously offered: (i) refined sugar or, (ii) refined sugar + hydrolyzed protein. Fly responses to latex droplets were assessed 4, 12 and 20 days after the start of the experiment. Ten female and ten male flies were selected at random from each cage and placed individually in transparent plastic cups (300 mL capacity). Each cup was placed upside down on a sheet of paper to prevent the escape of flies during handling. The cups had a small orifice located halfway up the cup which allowed a micropipette tip to be inserted into the cup. After a 15 min acclimatization period, a 3 µL droplet of fresh latex was deposited on the inner wall of the cup next to the orifice. Individual flies were continuously observed for 10 min. Two fly behaviors were recorded; (i) attraction, if the fly touched the latex drop with its proboscis, and (ii) consumption, if the fly remained with its proboscis in the latex for at least 10 s. The response time between introducing the latex and fly responses to the droplet was also recorded. Each group of 10 male and 10 female flies was considered to be a replicate. The experiment was performed for a total of three replicates of each diet and age combination.

### 2.7. Adult Longevity

To determine adult longevity under laboratory conditions, recently emerged groups of 10 male and 10 female flies were placed together in cages (3000 cm^3^ capacity) with a water source, and continuous access to refined sugar + hydrolyzed protein. A sheet of absorbent towel was placed in the base of each cage as a resting surface. The mortality of flies was registered at daily intervals until all insects had died. The procedure was performed with a total of 5 cages (replicates).

### 2.8. Statistical Analyses

The effect of treatment and age on the cross-sectional area of ovaries and testes (means of both ovaries or testes in each insect) was analyzed by fitting generalized linear models (GLMs) with a quasi-Poisson error distribution to account for overdispersion. The cross-sectional area was used as the dependent variable, with treatment and age as factors, and head width was included as a covariate serving as an indicator of adult body size. The prevalence (%) of sexually mature females on different diets, and with exposure to UV radiation and figs were analyzed by Fisher’s exact test. The percentage of male and female flies that responded to latex was analyzed by fitting a GLM with a binomial error distribution and a logit link function with treatment, sex and age as factors. The response time was analyzed by fitting a GLM with a quasi-Poisson distribution. The results of GLM analyses are presented as χ^2^ statistics. Means were compared by Bonferroni test. Adult longevity was estimated by fitting Kaplan–Meier survival curves and the sexes were compared by log-rank test. All analyses were performed using the R-based software Jamovi v. 2.3.28 [30].

## 3. Results

A total of 1842 adults of *S. adipata* (48% females, 52% males) emerged from sampled figs in the laboratory. Other dipterans were also present in these samples including lonchaeids (*Neosilba* spp.) and two exotic drosophilids, *Zaprionus indianus* (Gupta, 1970) and *Drosophila suzukii* (Matsumura, 1931), but these species were discarded and were not considered further.

### 3.1. Reproductive System of Silba adipata

#### 3.1.1. Females

**Figure 1:**
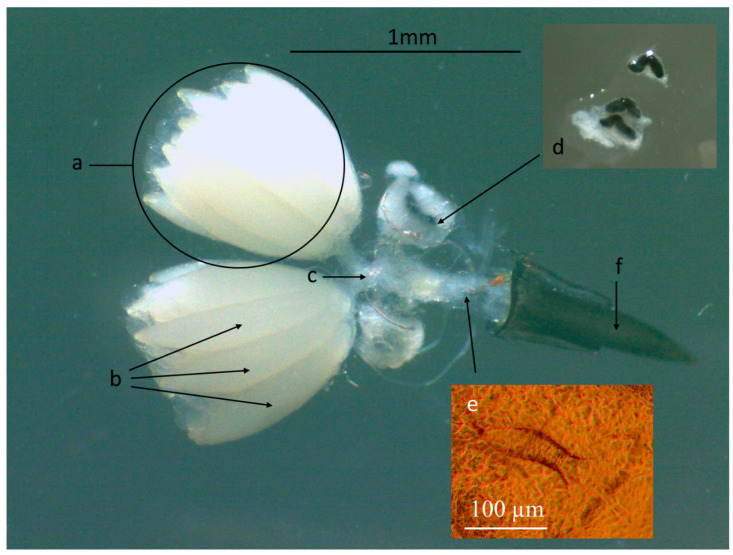
Reproductive system of a 21-d-old female *Silba adipata*, showing the following structures: ovary (a), oocytes (b), oviduct (c), spermathecae (magnified in inset image, (d)), signum (magnified in inset image, (e)) and ovipositor (f). The whitish outer cuticle of the spermathecae (d) was removed to observe their sigmoid structure.

Females of *S. adipata* have two ovoid-shaped translucent white ovaries with a mean (±SE) cross-sectional area of 0.767 ± 0.060 mm^2^ and are surrounded by grey filamentous tissue (Figure 1). Each ovary comprises an average of 11.12 ± 1.40 oocytes of mean length 862 ± 28 µm and width 214 ± 6 µm (labeled a in Figure 1). Each oocyte has a cross-section area of 0.142 ± 0.007 mm^2^. The oocytes are white, fusiform and are visible through the outer tissue of the ovary (labeled b in Figure 1). Oocytes connect to individual oviducts and then to a common oviduct (c in Figure 1). Females have three spermathecas located at the edges of the common oviduct, two of them paired and one placed individually (d in Figure 1). The spermathecas are black, sigmoidal and are covered by a whitish translucent spermathecal capsule. Within the common section of the oviduct, a sclerotized structure with a series of comb-like projections along its edges was identified. This structure appears to have an opening oriented toward the ovipositor. Based on its location and morphology, it is likely a signum (e in Figure 1). The signum is a sclerotized structure found in the female reproductive tract of certain arthropods that plays a significant role in reproduction, although its shape and size can vary considerably among species. The ovipositor measures 994 ± 58 µm in length and is lance shaped with an aperture that runs from the base to the lower tip (f in Figure 1). The ovipositor is black and covered by sclerotized chitin over its entire length.

#### 3.1.2. Males

**Figure 2:**
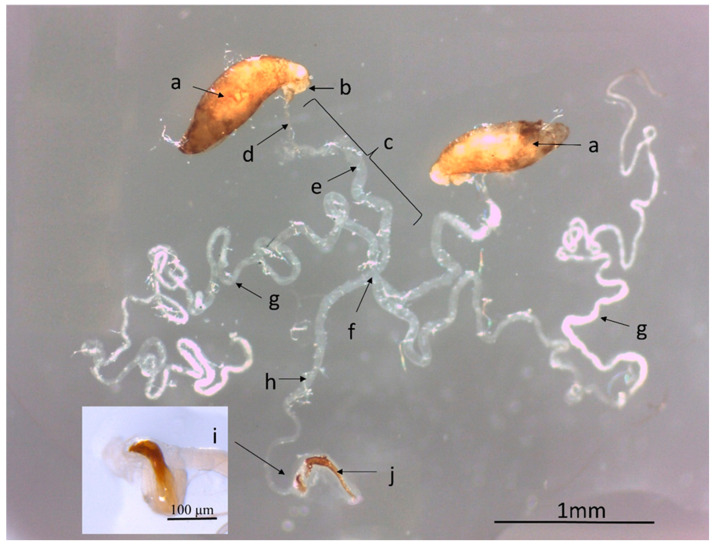
Reproductive system of a 21-d-old male *Silba adipata*, showing the following structures: testes (a), efferent duct (b), vas deferens (c), narrow duct of the vas deferens (d), wide duct of the vas deferens (e), common sperm duct (f), accessory glands (g), ejaculatory duct (h), ejaculatory bulb (magnified in inset image, (i)) and aedeagus (j).

The males of *S. adipata* have two fusiform yellow-brown testes of length 946 ± 20 µm and width 315 ± 10 µm with a mean cross-sectional area of 0.214 ± 0.007 mm^2^ (a in Figure 2). The testes are connected to the efferent ducts (labeled b in Figure 2), which are sigmoid-shaped and have the same color. The vas deferens comprise a narrow and a wider section (labeled d and e, respectively, in Figure 2) that connect the efferent ducts with the accessory glands (g in Figure 2) and the ejaculatory duct at the common sperm duct (f in Figure 2). The accessory glands consist of two elongated structures that are intertwined and have been separated in Figure 2 as far as possible to assist observation. In some individuals, the wide part of the deferent ducts appears whitish, suggesting it might fulfill the function of seminal vesicles; however, we were unable to verify the presence of sperm. The ejaculatory duct (h in Figure 2) is elongated and becomes narrower as it approaches the ejaculatory bulb (i in Figure 2). The ejaculatory bulb (mean cross-sectional area 0.016 ± 0.001 mm^2^) comprises white fibrous muscle tissue that surrounds the interior cavity of the brownish-yellow colored bulb (Figure 2). The aedeagus in this species is a simple structure with a slightly curved shape, measuring 565 ± 17 µm in length (j in Figure 2). It is connected to the ejaculatory bulb and is not retracted into the abdomen.

### 3.2. Effect of Sugar, Protein and Fig Latex Ingestion on Sexual Development

#### 3.2.1. Females

None of the female flies developed mature oocytes in the treatments involving sugar alone or sugar + fig latex over the 27 days of the experiment (Table 1). The immature oocytes in this species appear as small spheres, giving the ovary a lobular appearance, whereas the mature oocytes were much larger and fusiform (Supplemental Figure S1). A female was considered mature if it had at least two large oocytes (>800 µm in length) in the ovary. Sexually mature females were recorded after 15 d only for those diets that included hydrolyzed protein, with sugar or with sugar + fig latex. Notably, the prevalence of mature females increased to 33–56% in the treatments involving hydrolyzed protein at age 21–27 days but this difference was not significant (Fisher’s *p* = 0.1028) (Table 1).

**Figure 3:**
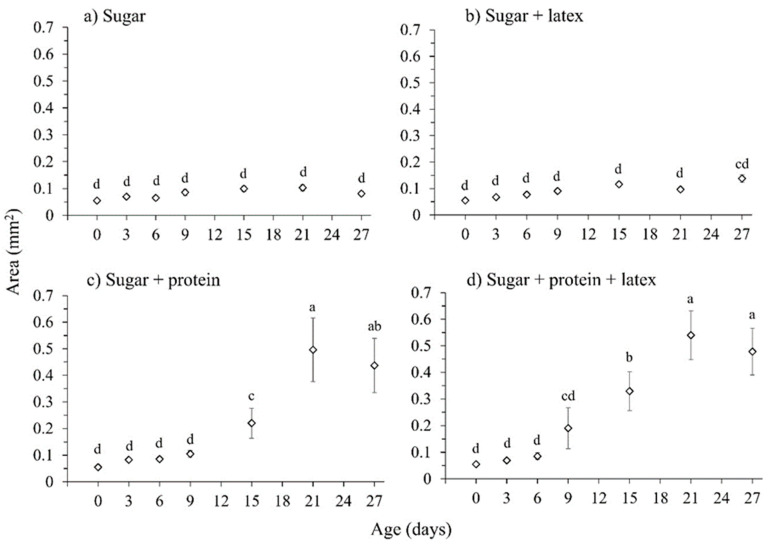
Mean area of both ovaries (±SE) in females aged 0, 3, 6, 9, 15, 21 and 27 d, that fed diets of (**a**) sugar, (**b**) sugar with latex, (**c**) sugar with protein and (**d**) sugar with protein and latex. Data points labeled with different letters differ significantly for comparisons among all diet treatments and female age (GLM, Bonferroni test, *p* < 0.05).

The cross-sectional area of the ovaries varied significantly with age (χ^2^ = 88.46, df = 6, *p* < 0.001) and diet (χ^2^ = 40.75, df = 3, *p* < 0.001) and with the interaction age × diet (χ^2^ = 56.09, df = 18, *p* < 0.001) (Figure 3). Ovary cross-sectional area also increased significantly with adult body size, measured by head width (χ^2^ = 7.88, df = 1, *p* = 0.005). Females fed sugar alone or sugar + fig latex treatments showed no significant changes in ovary size over time (Figure 3a,b). In contrast, ovary size increased significantly in females fed hydrolyzed protein with sugar and/or fig latex (Figure 3c,d). This increase started after 15 days, increased after 21 days and remained high at 27 days after the start of the experiment. This result highlighted the importance of protein in the sexual maturation of female flies.

#### 3.2.2. Males

**Figure 4:**
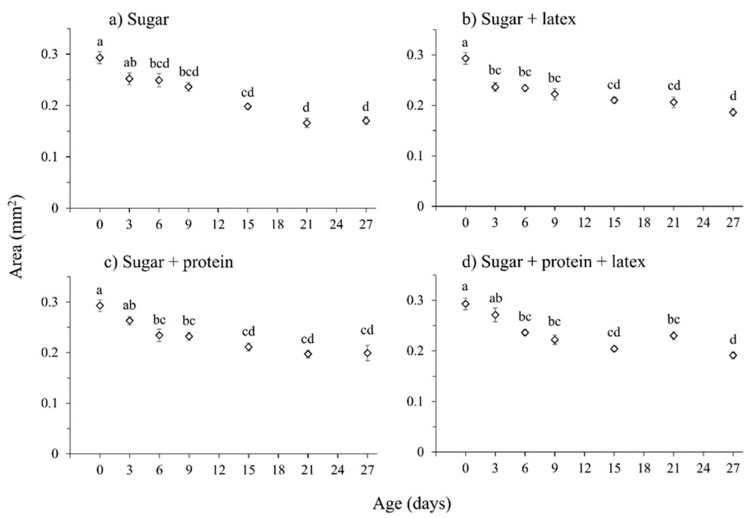
Mean area of both testes (±SE) in males aged 0, 3, 6, 9, 15, 21 and 27 d, fed diets of (**a**) sugar, (**b**) sugar with latex, (**c**) sugar with protein and (**d**) sugar with protein and latex. Data points labeled with different letters differ significantly for comparisons among all diet treatments and male age (GLM, Bonferroni test, *p* < 0.05).

The cross-sectional area of the testes varied significantly among dietary treatments (χ^2^ = 8.28, df = 3, *p* = 0.041), with the age of the male (χ^2^ = 322.25, df = 6, *p* < 0.001), and with the interaction of these factors (χ^2^ = 35.69, df = 18, *p* = 0.008) (Figure 4a–d). No significant relationship between testes size and male body size (head width) was detected (χ^2^ = 1.13, df = 1, *p* = 0.288). The males of *S. adipata* appear to emerge with well-developed testes that then continuously decreased in size as the flies age from 6 days after emergence.

### 3.3. Effects of Exposure to Ripe Fruit on Female Sexual Development

**Figure 5:**
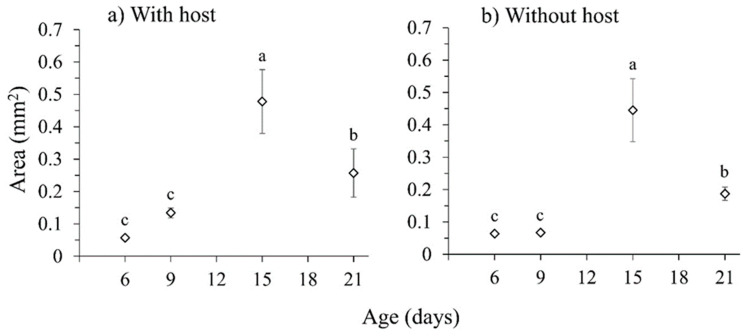
Mean ovarian area (±SE) at 6, 9, 15, and 21 d in the (**a**) presence and (**b**) absence of a fig host. Data points with different letters indicate significant differences for comparisons within each treatment (GLM, Bonferroni’s test, *p* < 0.05).

As observed previously (Section 3.2.1), ovary size increased significantly in females aged 15 days with continuous access to sugar + hydrolyzed protein when compared to females aged 6 and 9 days, and then decreased slightly in females aged 21 days (χ^2^ = 93.178, df = 3, *p* < 0.001) (Figure 5a,b). The presence of figs had no significant effect on ovary development (χ^2^ = 1.523, df = 1, *p* = 0.217) or on the age × host interaction (χ^2^ = 2.137, df = 3, *p* = 0.544). No significant relationship was detected between ovary size and female body size (head width) (χ^2^ = 0.083, df = 1, *p* = 0.773).

Sexually mature females were first observed at age 15 days (40–50% prevalence), either with or without host plant stimuli and the prevalence of mature females decreased significantly at age 21 days (Fisher’s *p* < 0.001) (Table 1). As an additional observation, some eggs were observed on the fruit surface or the cage wall at 21 days with or without fig stimuli, but none of the eggs were fertile.

### 3.4. Effect of Exposure to Ultraviolet Radiation on Ovary Development

**Figure 6:**
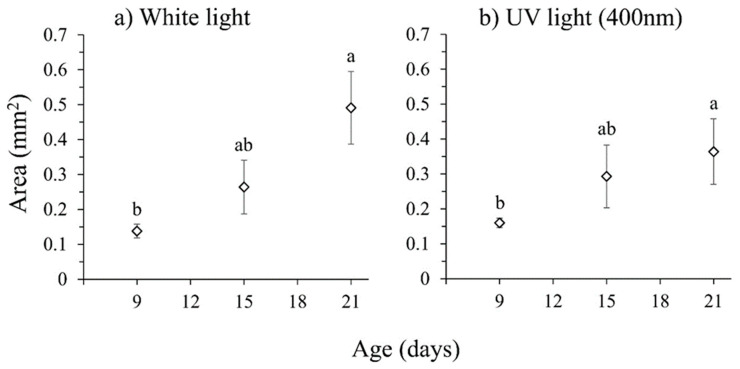
Mean ovarian area (±SE) in females of different ages reared under (**a**) white light and (**b**) white light supplemented with UV-A (400 nm). Data points with different letters indicate significant differences for comparisons within each treatment (GLM, Bonferroni’s test, *p* < 0.05).

As in the previous experiments, ovary development increased as the flies aged (χ^2^ = 13.772, df = 2, *p* = 0.001) in the continuous presence of sugar and hydrolyzed protein (Figure 6). However, the supplementation of white light with UV-A (400 nm) illumination did not influence ovary size (χ^2^ = 0.027, df = 1, *p* = 0.868) or the interaction of age × light (χ^2^ = 2.953, df = 2, *p* = 0.228) (Figure 6). No significant relationship between ovary size and female body size (head width) was detected in this experiment (χ^2^ = 1.192, df = 1, *p* = 0.275). Sexually mature females were first observed at age 15 days (10–20%), while the prevalence of mature females increased significantly (20–50% prevalence) at 21 days (Fisher’s *p* = 0.002) (Table 1).

### 3.5. Male and Female Responses to Fig Latex

The mean (±SE) response time of male and female flies to the latex droplet was 178 ± 15 s (*n* = 96 insects) and this did not differ significantly between the flies according to their sex (χ^2^ = 0.0249, df = 1, *p* = 0.875), age (χ^2^ = 0.218, df = 1, *p* = 0.64) or their previous diet (sugar vs. sugar + hydrolyzed protein) (χ^2^ = 0.487, df = 1, *p* = 0.485).

**Figure 7:**
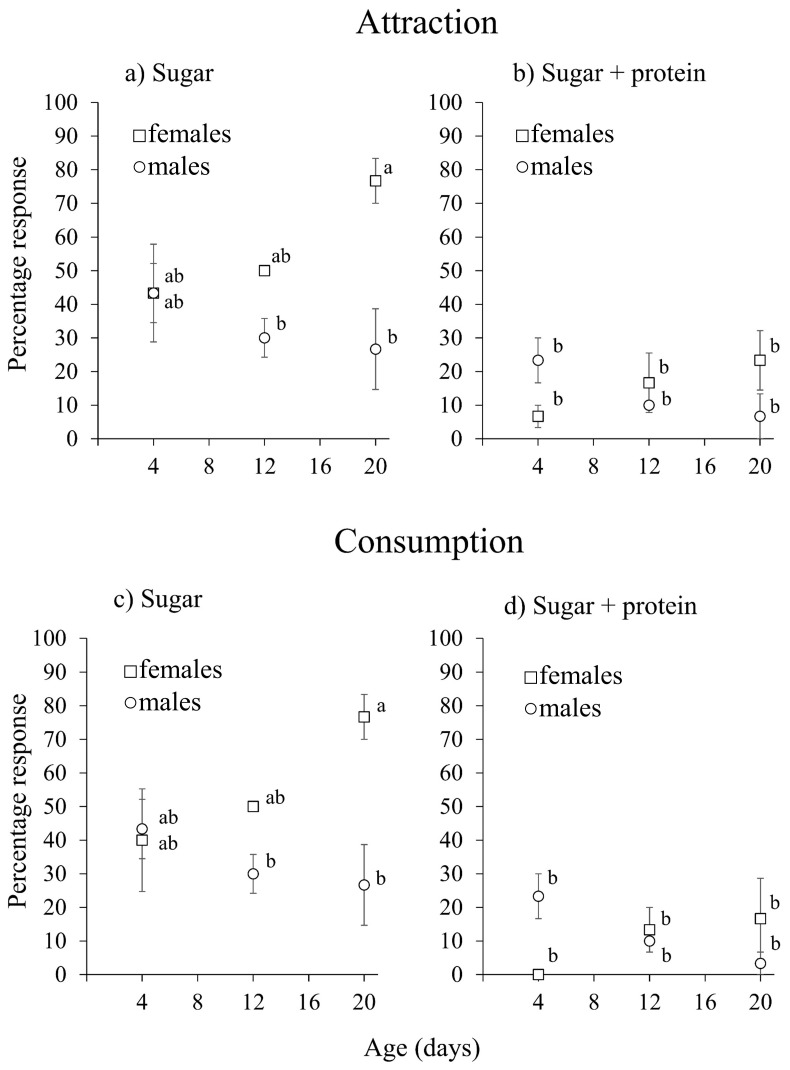
Mean percentage (±SE) of *Silba adipata* females and males attracted to and consuming latex at 4, 12 and 20 d of age after being previously fed sugar (**a**,**c**) or sugar + protein (**b**,**d**). Data points with different letters indicate significant differences for comparisons among diet treatments, sex and fly age (GLM, Bonferroni’s test, *p* < 0.05).

A significantly higher percentage of flies was attracted to fig latex when previously fed only sugar (45.0 ± 5.1%) than when fed on a mixture of sugar + hydrolyzed protein (14.4 ± 2.8%) (χ^2^ = 35.2, df = 1, *p* < 0.001) (Figure 7a,b), which did not vary significantly with the main effects of age (χ^2^ = 0.049, df = 2, *p* = 0.976) or sex (χ^2^ = 2.31, df = 1, *p* = 0.128). However, there was a significant interaction of age and sex (χ^2^ = 11.88, df = 2, *p* < 0.001), with a higher percentage of older females being attracted to latex whereas the attraction declined in older males (Figure 7a).

Almost all the flies that were attracted to the droplet (93.5%) also consumed the latex. Diet had a significant effect on latex consumption for both sexes (χ^2^ = 55.88, df = 1, *p* < 0.001) with a lower consumption in flies that were previously fed protein + sugar than sugar alone (Figure 7b). The percentage of flies that consumed the latex droplet did not differ between the sexes (χ^2^ = 0.089, df = 1, *p* = 0.765), or with age (χ^2^ = 2.336, df = 2, *p* = 0.311), but differed significantly with the interaction of sex × age (χ^2^ = 22.248, df = 2, *p* < 0.001) as consumption tended to increase with age in female flies but decreased with age in male flies (Figure 7b).

### 3.6. Longevity

**Figure 8:**
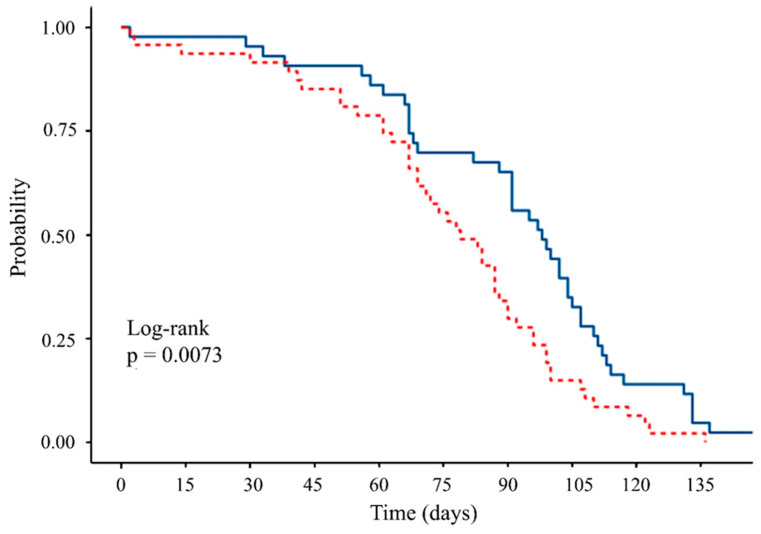
Survival probability of males (red) females (blue) of *Silba adipata* when reared in groups of 10 males and 10 females. The experiment was performed in 5 replicate cages.

A total of 43 females and 47 males were monitored across the five replicate cages for the duration of the experiment while offered a diet of sugar + hydrolyzed protein. Median survival time was lower for males (79 d, 95% CI = 69–90 d) than for females (98 d, 95% CI = 91–107 d) (Kaplan–Meier log-rank test, *p* = 0.007) (Figure 8).

## 4. Discussion

Understanding the life history, physiology and behavior of invasive pests, such as *S. adipata*, is key to the implementation of successful pest management programs [31]. In particular, information related to sexual maturation and sexual behavior are prerequisites for behavioral studies, which, together with additional information on the biology and ecology of the pest, help to define and improve control strategies [32].

Males and females of the same species, including insects, generally follow different growth and development trajectories that cause different sexes to reach sexual maturation at different ages [33]. The observed size of the testes in *S. adipata* males at emergence suggests that they are already sexually mature at this stage, as the testes reached their maximum size at emergence and progressively decreased with age, irrespective of the diet consumed. The males of some fly species of agricultural importance, such as *Anastrepha curvicauda* (Gerstaecker) (Diptera: Tephritidae) or *Delia planipalpis* (Stein) (Diptera: Anthomyiidae), emerge sexually mature, whereas females need several days to develop their ovaries [34,35]. In other species, such as *Bactrocera cacuminata* (Hering), the size of the ejaculatory bulb has been used as an indicator of sexual maturation instead of testis size. In *B. cacuminata*, the ejaculatory bulb grows with the age of males and reaches a maximum threshold that coincides with sexual maturity. In the present study, the size of the ejaculatory bulb was not evaluated, but it could be used in future research as a predictor of sexual maturity [36]. However, to confirm whether *S. adipata* males emerge sexually mature, it would be necessary to observe copulation following adult emergence, although this is challenging, as to date no observations of mating in this species have been recorded under laboratory conditions. Copulation was not observed during the experiments despite housing males together with mature females (15–27 days old) in the same cages. Unsuccessful attempts to reproduce this species in captivity have been reported previously [7] and may be related to the inability to replicate the specific conditions required for male swarming, which is essential for copulation in lonchaeid flies [37].

Unlike males, the sexual maturation of *S. adipata* females occurred gradually over a period of 15– 21 days and only took place when they consumed a diet supplemented with hydrolyzed protein. By age 15–21 days, the ovaries had reached their maximum development but subsequently decreased in size. This reduction in ovary size might be explained by the oviposition of unfertilized eggs, as eggs were observed on the cage walls during this period. Oviposition of non-fertile eggs was also previously documented for this species [7,9]. Based on the ovarian load of mature females, a *S. adipata* female can produce between 20 and 24 eggs, a significantly lower number than for other dipteran pests such as *Ceratitis capitata* Wiedemann (up to 1150 eggs) or *Anastrepha ludens* Loew (up to 1400 eggs) [17].

Females that consumed only carbohydrate failed to achieve sexual maturity, with their ovarian size remaining nearly unchanged from that observed at emergence. The positive effects of providing a combined diet of sugar and hydrolyzed protein have been extensively documented for tephritid flies [15,38]. The combination of hydrolyzed protein and sucrose resulted in a 27-fold increase in egg production in females of the Mediterranean fruit fly, *C. capitata* compared to flies that fed on sucrose alone [39]. Similar effects have been observed in other tephritid genera, such as *Anastrepha* and *Bactrocera* [12,40], and in the muscid, *Philornis downsi*, in which female diets supplemented with protein resulted in larger ovaries compared to females fed sugar alone [41].

*Silba adipata* has been monitored using traps baited with ammonium salts [7,23]. This attraction to ammonia in tephritid flies is often associated with their search for proteins to support sexual maturation [42,43,44]. Unlike protein, the inclusion of latex in the diet of *S. adipata* females did not significantly accelerate sexual maturation, perhaps due to its low protein content [45].

We found variation in the prevalence of females that reached sexual maturation at 15 days. For example, the percentage of control females that reached sexual maturity at 15 days varied from 40 to 50% in the experiment involving access to ripe figs compared to 8–20% of females in the diet experiment, or 12.5–20% in the UV-A exposure experiment. Similarly, the ovary size of 15-day-old females exposed to host fruit was comparable to that of 21-day-old females in the diet and UV-A trials. Clearly, these percentages provide only an initial estimate of sexual development given that they are based on modest sample sizes as the availability of flies in our experiments was dependent on the patterns of adult emergence from field-collected figs. Just over half of the females examined across all our experiments (40–56%) achieved an ovary size indicative of maturity at 15–21 days. This relatively low proportion of mature females suggests the existence of additional specific stimuli, not accounted for in our studies, that may play a pivotal role in facilitating sexual maturation. No significant differences were observed in the prevalence of sexually mature females or ovary size between females that had access, or not, to fig exudates indicating that fig exudates had no effect on sexual maturation in this fly. However, we cannot exclude the possibility that the early maturation of females in treatments with and without access to fig exudates may have been influenced by exposure to volatiles from figs in adjacent cages. The proximity of the cages in the laboratory was such that flies in cages without figs might have experienced the presence of volatiles emitted by the figs in the cages of the other treatment. This idea finds support from studies on the tephritid *Anastrepha obliqua* Macquart, in which the host fruit, or its chemical signals, stimulate egg maturation while simultaneously triggering oviposition [12].

Physiological processes and various behaviors in microbes, invertebrates and vertebrates have been reported to fluctuate according to the daily solar cycle and light intensity [46]. However, few studies on Diptera are available for comparison. In our study, UV-A (400 nm) light exposition did not enhance or accelerate the sexual maturation of *S. adipata* females. Most adult dipterans are thought to have color vision receptors sensitive to high-blue or low-green wavelengths (480–530 nm) [47], suggesting that other behavioral or physiological processes may be more influenced by this range of wavelengths. Zhang et al. [48] reached a similar conclusion in studies on the black soldier fly, *Hermetia illucens* (L.), in which light of 350–450 nm inhibited mating and oviposition, whereas broad spectrum light (350–2500 nm) did not. Light intensity is an additional factor that was not accounted for in our experiments. On a sunny day, the intensity of sunlight can reach 1 000 000 lux [49], a markedly higher light level than the illumination in our laboratory.

Females of *S. adipata*, had a median survival time of 98 days, but males had a lower median survival time of 79 days. Longevity and reproduction in insects are often closely correlated. In many tephritids, males typically have shorter lifespans due to the energetic costs and risks associated with their continual search for mating opportunities, whereas females often live longer to maximize their lifetime reproductive output [50]. Recently, Paniagua-Jasso et al. [9] indicated a lower adult survival time for *S. adipata* (around 45–55 d) that was similar for males and females that emerged from a different fig cultivar (Black Mission).

Although latex is not directly involved in sexual maturation, a high attraction was previously reported for adult flies of *S. adipata* [7,19,20], but this attraction was not quantified. Attraction of *S. adipata* to latex was modulated by sex, adult age and diet. In females, the proportion of individuals attracted to latex increased with age and peaked at age 20 d, which coincides with the typical onset of sexual maturity. By contrast, males exhibited higher levels of attraction to latex shortly after emergence, which gradually declined with age. In both sexes, the response to latex was higher when flies previously fed on sugar, but decreased if the flies had access to sugar and hydrolyzed protein.

The underlying reasons for attraction to latex in *S. adipata* are unclear. A greater attraction to this substance when flies were protein deprived could indicate that latex attraction is related to the need for protein. Latex is a chemically complex substance comprising organic acids, fatty acids, carbohydrates, amino acids and other nitrogenous compounds, along with a diverse array of volatile compounds [21,45,51]. Despite containing various amino acids, including leucine, tryptophan, serine, ornithine, tyrosine and cysteine [52], its overall protein content is relatively low [51]. The males of several species of tephritids of *Bactrocera* and *Ceratitis* are strongly attracted to specific plant compounds that act as pheromone precursors that improve sexual maturation and sexual behavior [53]. These pheromone precursors do not attract females although other plant materials, such as essential oils, have been reported to stimulate tephritid oviposition behavior [54].

## 5. Conclusions

This study advances our understanding of the physiology and reproductive biology of *S. adipata*, which now poses significant challenges for fig producers in Mexico and elsewhere. It provides the first detailed descriptions of the reproductive organs in both males and females and identifies a critical role of dietary proteins in the sexual development of females. This study also highlights the age-dependent responses of adults to latex and sex-specific differences in adult longevity. Future studies should focus on the key factors that lead to copulation and the production of fertile eggs, which would allow for the continuous culture of this invasive and little-studied pest.

## Figures and Tables

**Table 1:** Percentage of females showing development of oocytes at different ages (0–27 d). Data from three independent experiments that involved different diets, host and ultraviolet (UV) light treatments.

		Fly Age (Days)						
Experiment		0 d	3 d	6 d	9 d	15 d	21 d	27 d
**Diet**	Sugar alone	0%	0%	0%	0%	0%	0%	0%
		(*n* = 10)	(*n* = 10)	(*n* = 9)	(*n* = 10)	(*n* = 8)	(*n* = 9)	(*n* = 8)
	Sugar + fig latex	0%	0%	0%	0%	0%	0%	0%
		(*n* = 10)	(*n* = 8)	(*n* = 10)	(*n* = 11)	(*n* = 10)	(*n* = 8)	(*n* = 11)
	Sugar + hydrolyzed protein	0%	0%	0%	0%	8%	44%	33%
		(*n* = 10)	(*n* = 8)	(*n* = 8)	(*n* = 11)	(*n* = 13)	(*n* = 9)	(*n* = 12)
	Sugar + hydrolyzed protein + fig latex	0%	0%	0%	0%	20%	46%	56%
		(*n* = 10)	(*n* = 8)	(*n* = 10)	(*n* = 10)	(*n* = 15)	(*n* = 13)	(*n* = 9)
**Host**	Fig host (sugar + hydrolyzed protein)			0%	0%	50%	20%	
				(*n* = 10)	(*n* = 8)	(*n* = 10)	(*n* = 10)	
	Without fig host (sugar + hydrolyzed protein)			0%	0%	40%	0%	
				(*n* = 10)	(*n* = 10)	(*n* = 10)	(*n* = 10)	
**UV**	UV light (sugar + hydrolyzed protein)				0%	20%	20%	
					(*n* = 10)	(*n* = 10)	(*n* = 10)	
	Without UV light (sugar + hydrolyzed protein)				0%	12.5%	50%	
					(*n* = 10)	(*n* = 8)	(*n* = 10)	

*n* = numbers of insects dissected in each treatment.

## Data Availability

The original contributions presented in this study are included in the article and in Supplementary File_S1.xlsx. Further inquiries can be directed to the corresponding authors.

## Supplementary Materials

The following supporting information can be downloaded at: https://www.mdpi.com/article/10.3390/insects16050495/s1, raw data file, File_S1.xlsx; Figure S1: Ovary size and oocyte development in *Silba adipata* females of different ages with access to sugar and hydrolyzed protein.
