# Supplementary material for: Sexual Development of Silba adipata (Diptera: Lonchaeidae): Effects of Diet, Ultraviolet Light and Fig Latex
Source: Insects. 2025 May 5;16(5):495. doi: 10.3390/insects16050495 (PMC12111942; doi:10.3390/insects16050495)
Supplement: Supplementary file 1 [file insects-16-00495-s001.zip › Figure S1.pdf]

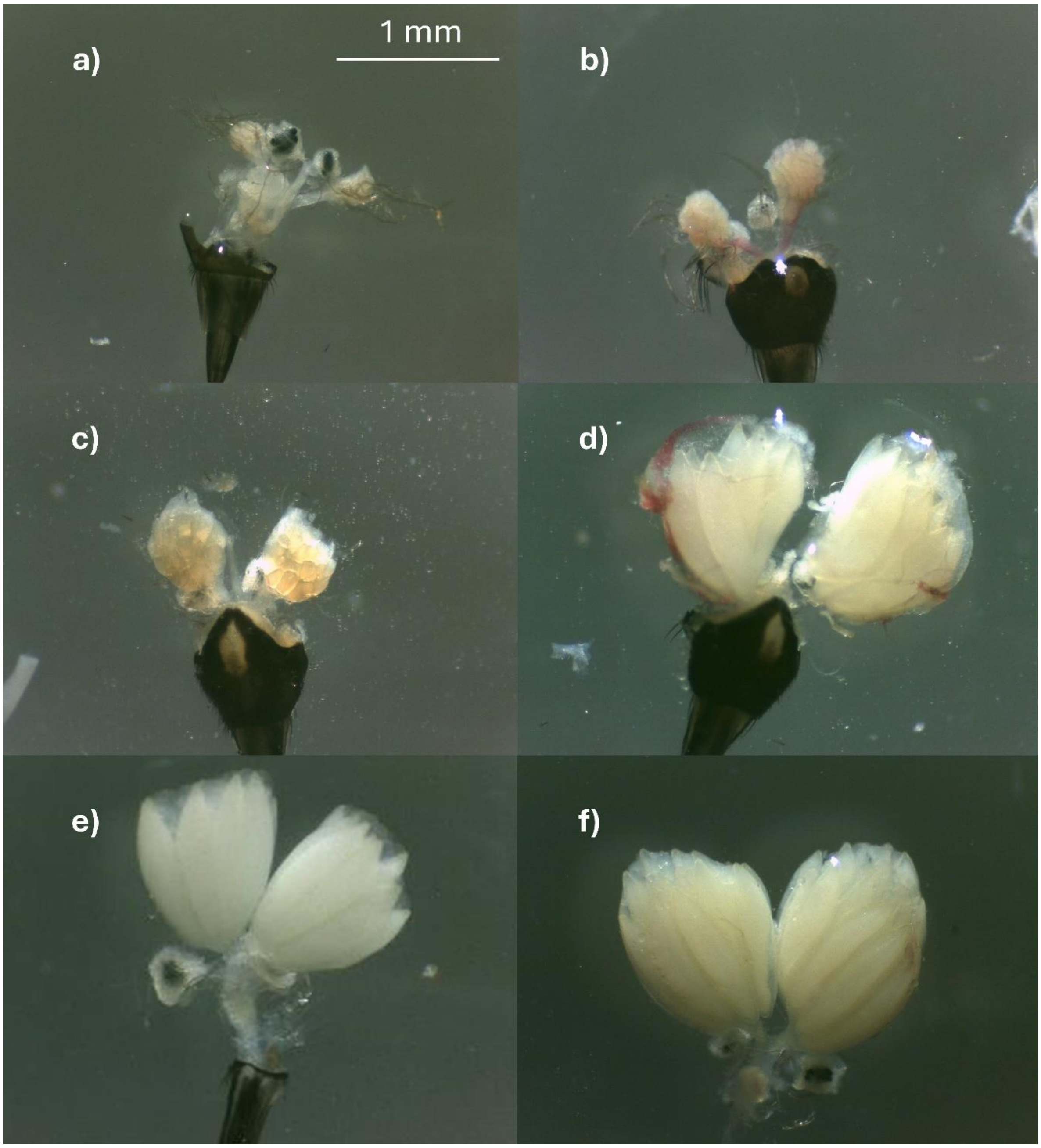

Figure S1. Ovary size and oocyte development in *Silba adipata* females of different ages with access to sugar and hydrolyzed protein. Development proceeded from: a) immature 6-day-old female, b) immature 9- day-old female, c) immature 15-day-old female, d) mature 15- day-old female, e) mature 21-day-old female, f) mature 27-day-old female.
